# Targeting the CXCR4 pathway using a novel anti-CXCR4 IgG1 antibody (PF-06747143) in chronic lymphocytic leukemia

**DOI:** 10.1186/s13045-017-0435-x

**Published:** 2017-05-19

**Authors:** Manoj K. Kashyap, Carlos I. Amaya-Chanaga, Deepak Kumar, Brett Simmons, Nanni Huser, Yin Gu, Max Hallin, Kevin Lindquist, Rolla Yafawi, Michael Y. Choi, Ale-Ali Amine, Laura Z. Rassenti, Cathy Zhang, Shu-Hui Liu, Tod Smeal, Valeria R. Fantin, Thomas J. Kipps, Flavia Pernasetti, Januario E. Castro

**Affiliations:** 1 Moores Cancer Center, University of California San Diego, 3855 Health Science Drive, La Jolla, CA 92093-0820 USA; 2 CLL Research Consortium, and Department of Medicine, University of California San Diego, La Jolla, CA USA; 3 Oncology Research & Development, Pfizer Worldwide Research & Development, 10646 Science Center Drive, San Diego, CA 92121 USA; 4 Oncology Research & Development—Rinat Biotechnology Unit, Pfizer Worldwide Research & Development, South San Francisco, CA USA; 5 Drug Safety Research & Development, Pfizer Worldwide Research & Development, La Jolla, CA USA; 6 Present Address: Eli Lilly and Company, Lilly Corporate Center, Indianapolis, IN USA; 7 Present Address: ORIC Pharmaceuticals, South San Francisco, CA USA; 8 Present Address: Mirati Therapeutics, San Diego, CA USA

**Keywords:** Chronic lymphocytic leukemia, PF-06747143, CXCR4, CXCL12, Chemokine, ADCC, CDC, Cell death, Reactive oxygen species

## Abstract

**Background:**

The CXCR4-CXCL12 axis plays an important role in the chronic lymphocytic leukemia (CLL)-microenvironment interaction. Overexpression of CXCR4 has been reported in different hematological malignancies including CLL. Binding of the pro-survival chemokine CXCL12 with its cognate receptor CXCR4 induces cell migration. CXCL12/CXCR4 signaling axis promotes cell survival and proliferation and may contribute to the tropism of leukemia cells towards lymphoid tissues and bone marrow. Therefore, we hypothesized that targeting CXCR4 with an IgG1 antibody, PF-06747143, may constitute an effective therapeutic approach for CLL.

**Methods:**

Patient-derived primary CLL-B cells were assessed for cytotoxicity in an in vitro model of CLL microenvironment. PF-06747143 was analyzed for cell death induction and for its potential to interfere with the chemokine CXCL12-induced mechanisms, including migration and F-actin polymerization. PF-06747143 in vivo efficacy was determined in a CLL murine xenograft tumor model.

**Results:**

PF-06747143, a novel-humanized IgG1 CXCR4 antagonist antibody, induced cell death of patient-derived primary CLL-B cells, in presence or absence of stromal cells. Moreover, cell death induction by the antibody was independent of CLL high-risk prognostic markers. The cell death mechanism was dependent on CXCR4 expression, required antibody bivalency, involved reactive oxygen species production, and did not require caspase activation, all characteristics reminiscent of programmed cell death (PCD). PF-06747143 also induced potent B-CLL cytotoxicity via Fc-driven antibody-dependent cell-mediated cytotoxicity (ADCC) and complement-dependent cytotoxicity activity (CDC). PF-06747143 had significant combinatorial effect with standard of care (SOC) agents in B-CLL treatment, including rituximab, fludarabine (F-ara-A), ibrutinib, and bendamustine. In a CLL xenograft model, PF-06747143 decreased tumor burden and improved survival as a monotherapy, and in combination with bendamustine.

**Conclusions:**

We show evidence that PF-06747143 has biological activity in CLL primary cells, supporting a rationale for evaluation of PF-06747143 for the treatment of CLL patients.

**Electronic supplementary material:**

The online version of this article (doi:10.1186/s13045-017-0435-x) contains supplementary material, which is available to authorized users.

## Background

CXCR4 (chemokine C-X-C motif receptor 4), also known as CD184, is a chemokine G protein coupled receptor [1], expressed in different cell types, including normal B cells [2–6]. CXCR4 is overexpressed in a variety of cancers including chronic lymphocytic leukemia (CLL), acute myeloid leukemia (AML), myeloma, lymphomas, and solid tumors [7]. CXCL12 (chemokine C-X-C motif ligand 12), also known as stromal cell-derived factor 1 (SDF-1), is CXCR4 sole ligand. It is a homeostatic chemokine [8], highly expressed in the lymph nodes, bone marrow (BM), liver, and lung [9]. CXCL12 regulates hematopoietic cell trafficking and their homing to the BM [5]. Chemotaxis driven by CXCR4 and CXCL12 interactions has been shown to control various biological functions including cell adhesion, migration, and invasion [10].

CLL is the most prevalent adult leukemia and is characterized by accumulation of dysfunctional B-lymphocytes in the lymph nodes and BM [11]. Stromal cells secrete CXCL12 and promote B-cell progenitors and CLL cell survival through CXCR4 signaling [8, 12, 13]. Thus, activation of the CXCL12/CXCR4 axis plays an important role in stromal cell-dependent resistance to therapy in CLL patients, including cytotoxic drugs [4] or steroids [14], thereby promoting minimal residual disease [15]. These observations support the rationale for targeting CXCR4 for the treatment of CLL.

In the last decade, a number of agents targeting CXCR4 have been developed. These include small molecules, peptides, and monoclonal antibodies. The role of CXCR4 in hematopoietic stem cell (HSC) retention and trafficking led to the development of agents used for HSC mobilization. AMD3100 (Plerixafor), a small molecule inhibitor of CXCR4, was approved for mobilization of HSCs prior to autologous transplantation; however, this compound has limited application for sustained treatment due to toxicity [16, 17]. BL-8040 (BKT140), a peptide inhibitor of CXCR4, has robust cell mobilization capacity [18, 19], similarly to other CXCR4-specific antagonist peptides (T-140, TN-14003, TC-14012), which were shown to inhibit CXCR4-CXCL12 signaling in CLL-B cells [4]. However, these peptides show limited in vivo exposures. Recently, two CXCR4 human IgG4 antagonist antibodies, ulocuplumab [20–22] and LY2624587 [23], were described. Ulocuplumab is currently in phase 1 clinical studies, and it was shown to have prolonged pharmacokinetic exposure compared to small molecules or peptide inhibitors [20–22].

PF-06747143 is a novel and potentially first in class humanized IgG1 anti-CXCR4 antibody that recently entered into clinical studies (NCT02954653). Here, we show that it potently binds to CXCR4 and inhibits CXCL12-driven calcium flux. Moreover, it induces cell death in malignant CLL-B cells via two main mechanisms of action: (1) bivalency-dependent mechanism, involving generation of reactive oxygen species (ROS) and independent of caspases and (2) Fc region-driven cytotoxicity, including complement-dependent cytotoxicity (CDC) and antibody-dependent cell-mediated cytotoxicity (ADCC) activity. Importantly, we show that PF-06747143 triggers cell death in B-CLL patient-derived primary leukemia cells, in spite of the presence of stromal cells, mimicking the leukemia microenvironment in vitro. The antibody also synergizes with conventional CLL treatment agents such as bendamustine, rituximab, fludarabine (F-ara-A), and ibrutinib, significantly improving their cytotoxicity in combination. Furthermore, we show that PF-06747143 inhibits tumor burden and improves survival as a monotherapy or in combination with bendamustine, in a CLL xenograft tumor model. Based on these unique mechanisms of action, PF-06747143 has a promising therapeutic potential in CLL patients and other hematological malignancies dependent on the CXCR4 axis.

## Methods

### Isolation of PBMCs from CLL patients

The CLL-B cells were collected from blood samples at the Moores-UCSD Cancer Center in compliance with the Declaration of Helsinki and after approval of the UC San Diego Institutional Review Board (IRB) [24].Peripheral blood mononuclear cells (PBMC) from CLL patients were isolated using Ficoll-Hypaque gradient density centrifugation (Cat# 17-1440-03, GE Healthcare Life Science). For caspase activation assays, the CLL-B cells were purified by positive selection using Dynabeads CD19 pan B (Cat# 11143D, Invitrogen) and DETACHaBEAD CD19 (Cat# 12506D, Invitrogen) according to the manufacturer’s protocol. For the other assays, fresh or frozen PBMCs were used and cells were stained with CD19/CD5 antibodies for detection of double positive CLL-B cells.

### CLL-B cells co-culture to mimic CLL microenvironment

Primary leukemic cells from CLL patients were cultured in RPMI supplemented with 10% heat-inactivated FBS (fetal bovine serum, Catalog # FB-02, Omega Scientific, Tarzana, CA) and 1% antibiotic at a density of 3 × 10^5^ cells per milliliter at 37 °C and 5% CO_2_. The cells were either cultured in 96-well round bottom plates (Catalog # 3596, Corning, NY) alone or co-cultured with NK-tert stromal cells (RIKEN, Yokohama, Japan) at a ratio of 20:1 (CLL: stroma-NK-tert) in RPMI with 1% Penn-Strep and 10% FBS [25].

### CXCR4 expression by flow cytometry

The CXCR4 phenotyping of CLL-B, stroma-NK-tert, normal B, and T cells was done by flow cytometry using a 1:50 dilution with rat anti-human CD184 (CXCR4) PE Mab (Catalog # 551966, clone:2B11, BD Biosciences). The isotype control antibody was PE Rat IgG_2b_, κ (Catalog # 12-4031-83, clone: eB149/10H5, eBioscience).

### CXCR4 antibody generation

The parental CXCR4 antagonist antibody, m15, was derived from immunization of Balb/c mice with CHO cells transfected with human CXCR4. The heavy and light chain variable domains of m15 were then cloned into human IgG1 or hinge stabilized IgG4 and light κ backbone, to generate chimeric m15-IgG1 and m15-IgG4. m15 was subsequently humanized by CDR grafting/affinity maturation and cloned into human IgG1/κ constant domains to create PF-06747143.

### Binding kinetics and affinity

Experiments were performed on a Biacore^TM^ T200 surface plasmon resonance biosensor (GE Life Sciences). The binding to human CXCR4 was determined using human CXCR4-enriched lipoparticles (Integral Molecular) compared to null particles. Lipoparticles were diluted into 10 mM HEPES, 150 mM NaCl, 1 mg/mL BSA, pH 7.4 buffer to concentrations between 0.015 to 0.04 units/mL and captured for 5 min onto flow cells. A threefold dilution series of Fab was evaluated and dissociation was monitored for 10 min. The data were fit to a 1:1 Langmuir with mass transport model using Biacore T200 Evaluation Software Version 2.0.

### PF-06747143 binding to tumor cells by flow cytometry

Cell suspensions (*n* = 3/group) were stained with 20 μg/mL of either a human IgG1 ĸ Phycoerthrin (PE)-labeled antibody (isotype control) (Southern Biotech) or with PF-06747143 PE-conjugated antibody, labeled using the SiteClick™ R-PE Kit (Molecular Probes, Life Technologies). Flow cytometric acquisition and analysis was conducted using FACS LSRII™ flow cytometer (Beckman Dickinson).

### Calcium flux functional assay

The ability of PF-06747143 or m15-IgG1 to inhibit CXCL12-induced calcium flux was evaluated in human T cell leukemia Jurkat cells using the Fluo-NW Calcium assay kit (Life Technologies). Cells were plated in 384-well plates at 70,000 cells per well in quadruplicates and incubated with m15-IgG1 parent antibody and PF-06747143, upon stimulation with CXCL12 at 8 nM (EC80) (Invitrogen), for 110 min. Calcium flux was then measured for 95 s using a FLIPR Tetra (Molecular Devices).

### Cell death

Cell death was evaluated by flow cytometry analysis using CD19/CD5/Annexin V antibodies [26]. Specific induced cell death (SICD) calculation was used in order to discriminate the antibody/compound-specific induced cell death from background or spontaneous cell death observed in the vehicle-treated groups. The calculation of % SICD was performed using the following formula: % SICD = (Compound-induced cell death − Vehicle spontaneous cell death)/(100 − Vehicle spontaneous cell death) × 100.

### Cell death in combination with CLL standard of care agents

m15-IgG1 was tested in combination with different standard of care (SOC) agents currently used for treatment of CLL. F-ara-A, bendamustine, rituximab, and ibrutinib were evaluated in combination with 200 nM of m15-IgG1. CLL-B cells were treated for 48 h at 37 °C either cultured alone or co-cultured with stroma-NK-tert cells. The combination data and level of synergism was analyzed using CompuSyn software (ComboSyn, Inc., NJ, USA). The data derived from this analysis were expressed as combination index (CI), which offers definition for additive (CI = 1), synergism (CI < 1), and antagonism (CI > 1) in drug combination [27].

### Antibody-dependent cellular cytotoxicity (ADCC) assay

For analysis of ADCC in B-CLL patient primary cells, the ADCC Reporter Bioassay kit from Promega (Catalog #G7010) was used, per instructions from the manufacturer. The ADCC Reporter Bioassay uses engineered Jurkat cells stably expressing the FcγRIIIa receptor, V158 (high affinity) variant, and a NFAT (nuclear factor of activated T cells) pathway response element driving expression of firefly luciferase as effector cells. The transfected Jurkat cell line was grown in RPMI containing G-418 sulfate solution (Catalog # V8091) and hygromycin (Catalog # 10687010, 50 mg/mL solution). The ADCC buffer (99.5% RPMI 1640 with L-glutamine and 0.5% super low IgG FBS) was prepared using RPMI supplemented with super low IgG defined fetal bovine serum (catalog # SH30898, Hyclone). The luciferase assay system was used as a readout (Catalog # G7940, Promega). Different concentrations of antibodies IgG1 control, PF-06747143, rituximab and obinutuzumab were added to the effector/target cell 1:1 ratio mixtures. A total of 75,000 for effector and target cells were incubated for 6 h at 37 °C in a humidified CO_2_ incubator. Following incubation, the plate was equilibrated to ambient temperature for 15 min. Bio-Glo™ luciferase assay reagent was added and incubated at room temperature for 30 min. The luminescence was detected using an Infinite 200 Microplate Reader (Tekan), and the results are expressed in relative light units (RLU).

ADCC activity of PF-06747143 and m15-IgG1 parent antibody was evaluated in JVM-13 CLL tumor cell line, in presence of the NK-92 FcγRIIIA 158V (NK92 158V) cell line as effector cells (Conkwest). Antibodies were incubated for 4 h, with tumor cells and effector cells (1:10 ratio) (*n* = 4/group). ToxiLight bioluminescent cytotoxicity assay (Cat # LT07-117 Lonza) was used to detect cell lysis.

### CDC assay

PF-06747143 was added to CLL-B cells (1 × 10^6^/mL) in RPMI media with 5% active human serum [28, 29] or inactivated human serum, which was incubated at 56 °C for 30 min. The heat-inactivated/normal human serum-treated cells were incubated for 4 h at 37 °C with increasing concentrations of PF-06747143. Cytotoxicity was determined by flow cytometry using CD19/CD5/Annexin V staining. % SICD was calculated according to the following formula: 100 × (% viable cells with inactivated serum − % viable cells with native serum)/(% viable cells with inactivated serum).

### Inhibition of actin polymerization

Cytoskeletal reorganization (F-actin polymerization) was evaluated in CLL samples activated by CXCL12 and treated with PF-06747143 or control agents [4].

### Inhibition of migration of cells in a transwell assay

PF-06747143 was assessed for its ability to inhibit CXCL12-induced chemotaxis in primary CLL-B cells derived from CLL patients using a transwell migration assay [30].

### Caspase activity assay

To evaluate the mechanism of cell death induced by PF-06747143, CLL-B cells were purified from patient-derived PBMCs and tested for caspase activation including caspases 3, 8, and 9 using the ApoTarget Caspase Colorimetric Protease Assay Sampler kit (Cat # KHZ1001, Invitrogen, Frederick, MD) according to the manufacturer’s instructions. Z-VAD-FMK, a caspase inhibitor (Cat # G7231, Promega Corporation), was used as control [31].

### Detection of reactive oxygen species (ROS) by flow cytometry

CLL-B cells were seeded at 2.5 × 10^5^/mL in RPMI media and treated with antibodies for 4 h at 37 °C and 5% CO_2_ in 24-well plates. The generation of ROS was detected using dihydroethidium (HE) staining (Catalog # D1168, Sigma-Aldrich, St. Louis, MO) as described previously [32]. The samples were then analyzed by flow cytometry followed by data analysis using FlowJo software.

### In vivo efficacy study

JVM-13 tumor cell line [33, 34], purchased from ATCC, was stably transfected with the luciferase gene. The cells were cultured in RPMI media with 10% FBS. To establish a JVM-13 disseminated model, 1 × 10^6^ cells per mouse were implanted via tail vein injection in female SCID beige mice (Charles River). Tumor burden was monitored via bioluminescence imaging (BLI) (IVISÒ 200) throughout the study. When the tumor burden (mean BLI) reached 7.2 × 10^6^ photons/s, on day 19, mice were randomly assigned into four groups and treated with (1) IgG1 negative control Ab or (2) PF-06747143, dosed subcutaneously at 10 mg/kg, once a week, for a total of 6 doses; (3) bendamustine, dosed intraperitoneally at 30 mg/kg, on days 19 and 20, followed by another 2-day treatment cycle 28 days later; and (4) combination of PF-06747143 and bendamustine. Mice were euthanized according to the IACUC guidelines once they developed disease-related symptoms such as hind leg paralysis.

### Statistical analysis

The statistical analysis was carried out using GraphPad Prism software (v. 5.0c; San Diego, CA). The statistical differences for the mean values were analyzed using one way ANOVA and are indicated with *, *p* < 0.05; **, *p* < 0.01; ***, *p* < 0.001; and ****, *p* < 0.0001. Tumor model survival analysis was performed using Kaplan-Meier followed by a long-rank (Mantel-Cox) test.

## Results

### Expression of CXCR4 in CLL, normal B cells, stroma-NK-tert, leukemia, and lymphoma cell lines

**Fig. 1:**
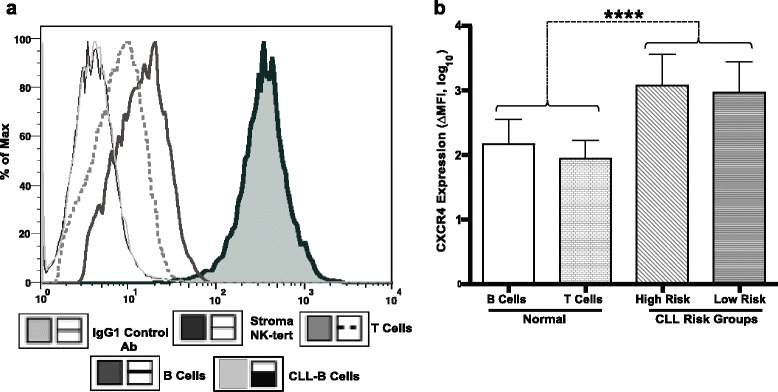
Expression of CXCR4 receptor in CLL-B, normal B, T, and stroma-NK-tert cells. **a** CXCR4 expression was assessed by surface staining using an anti-CXCR4 antibody in CLL-B, normal B, T, and stroma-NK-tert cells. A representative panel is shown. **b** CXCR4 expression was evaluated by flow cytometry in CLL patient cells with high-risk and low-risk characteristics (*n* = 20 per group) and in normal B and T cells obtained from healthy donors (*n* = 5 per group). The figure shows the mean fluorescence intensities (MFI) ± standard deviation (SD) of the samples analyzed in duplicate for CXCR4 expression in each cell type. Statistical significance was determined by using Bonferroni’s correction test for multiple comparison tests, where *, **, ***, and **** represent *p* < 0.05; *p* < 0.01, *p* < 0.001, and *p* < 0.0001, respectively

Expression of CXCR4 was evaluated by flow cytometry in primary B-CLL cells from patients, as well as normal B, T, and stroma-NK-tert cells. A representative example of CXCR4 staining is shown in Fig. 1a. CXCR4 expression was higher in CLL-B cells compared to normal B and T cells. We then assessed CXCR4 expression levels in high- and low-risk CLL patient groups. CLL patients were stratified into high risk (ZAP-70 ≥ 20%, IgVH ≥ 98% homology) and low risk (ZAP-70 < 20%, IgVH < 98% homology) based on ZAP-70 expression and IgVH homology status [35]. The average mean fluorescence (ΔMFI) for CXCR4 expression in CLL samples ranged from 263.73 to 2401.7, regardless of high- or low-risk status. The ΔMFI in high-risk patients was 1497.23 ± 195.89 and in low-risk patients, it was 1533.73 ± 178.54. Upon comparison between low- and high-risk CLL patients, no significant difference in CXCR4 expression levels was observed (*p* = 0.8601). Overall, CXCR4 expression was significantly higher in CLL-B cells as compared to normal B or T cells (10- and 22-fold, respectively; *p* < 0.0001) (Fig. 1b). Furthermore, CXCR4 expression in leukemia and lymphoma cell lines including K562, MEC1, Namalwa, Raji, JeKo-1, and Jurkat showed a broad range of expression levels, with ΔMFIs of −2.35, 0.01, 73.41, 526.72, 725.45, and 1358.5, respectively (Additional file 1: Figure S1).

### PF-06747143 and the parental m15 antibody bind CXCR4 with high selectivity and affinity

**Fig. 2:**
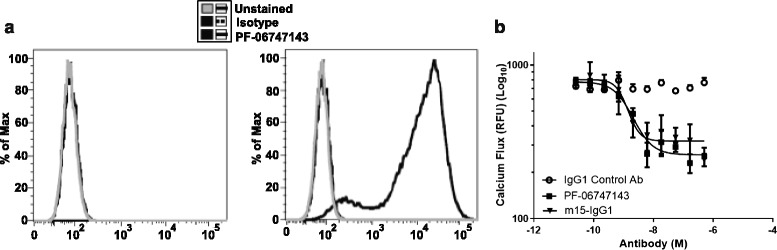
PF-06747143 binds specifically to human CXCR4-expressing cells and blocks CXCL12-induced calcium flux. **a** CHO-parental and CHO-hCXCR4 cell lines were exposed to 20 μg/mL of either a human IgG1 ĸ-PE antibody (isotype control) or PF-06747143-PE and analyzed by flow cytometry. **b** Calcium flux assay was performed in human T cell leukemia Jurkat cells incubated with PF-06747143, m15-IgG1, or isotype control IgG1 antibody in presence of CXCL12 at 8 nM. Experiment was performed in quadruplicates. Shown are mean intracellular calcium concentrations in relative fluorescence units (RFU). ± standard error of the mean (SEM)

To determine the binding specificity and affinity of PF-06747143 and its parental antibody m15 to human (h) CXCR4, we employed surface plasmon resonance. The monovalent Fab fragments of each antibody were incubated with hCXCR4-enriched lipoparticles. The apparent equilibrium dissociation constant (*K*_*D*_) was 0.36 nM for PF-06747143 and 0.67 nM for the parental antibody, m15, indicating that both antibodies have potent and comparable affinity to hCXCR4 (Table 1). None of the Fabs bound to lipoparticles lacking hCXCR4 (data not shown). In a separate experiment, PF-06747143 was fluorescently labeled (PF-06747143-PE) and binding to CHO cells expressing hCXCR4 (CHO-hCXCR4) was compared to binding to cells transfected with empty vector (CHO-parental). PF-06747143-PE bound specifically to CHO cells expressing hCXCR4 (CHO-hCXCR4) but not to the CHO-parental cells (Fig. 2a), demonstrating selective binding to CXCR4-expressing cells.

**Table 1:** Biosensor kinetics and affinity measurements

Clone name	*k*_*a*_ (1/Ms)	*k*_*d*_ (1/s)	*t*_1/2_ (min)	*K*_*D*_ (nM)
m15 Fab	2.1 × 10^6^	1.4 × 10^−3^	8.3	0.67
PF-06747143 Fab	8.0 × 10^5^	2.9 × 10^−4^	40	0.36

Binding affinities of the Fab regions derived from PF-06747143 and its parent antibody m15 were measured by surface plasmon resonance

*k*_*a*_ kinetic association constant, *k*_*d*_ kinetic dissociation constant, *t*_1/2_ dissociation half-life, *K*_*D*_ equilibrium dissociation constant

### PF-06747143 and the parental antibody m15-IgG1 inhibit CXCL12-induced calcium flux

Calcium flux is triggered upon activation of CXCR4 by its ligand, CXCL12. We next evaluated the ability of PF-06747143 and its parental antibody, m15, expressed as a chimeric human IgG1 antibody (m15-IgG1), to inhibit calcium flux induced by CXCL12. The Jurkat T cell leukemia line, which expresses high levels of CXCR4 (Additional file 1: Figure S1), was incubated with CXCL12 (EC_80_ at 8 nM) to stimulate calcium flux. A titration of PF-06747143 and m15-IgG1 was performed. Both PF-06747143 and m15-IgG1 blocked CXCL12-induced calcium flux in a dose-dependent manner, with similar IC_50s_ of 1.41 and 1.13 nM, for PF-06747143 and m15-IgG1, respectively. These results show that both CXCR4 antibodies have potent and comparable CXCL12 antagonistic activity (Fig. 2b).

Next, we evaluated if bivalency was required for PF-06747143 to inhibit calcium flux. To this end, a bivalent form of PF-06747143, which has no constant Fc region [F(ab’)_2_], and a monovalent form of the antibody [Fab], were generated and compared to PF-06747143, which is a bivalent full-length antibody (PF-06747143 FL). (Additional file 2: Figure S2). Similar CXCL12-induced calcium flux inhibition was observed for all three forms of PF-06747143 tested, indicating that the functional CXCL12 antagonistic activity is not dependent on bivalent binding or Fc constant region of the antibody.

### The CXCR4 antibody induces cell death in CXCR4-expressing CLL patient cells

**Fig. 3:**
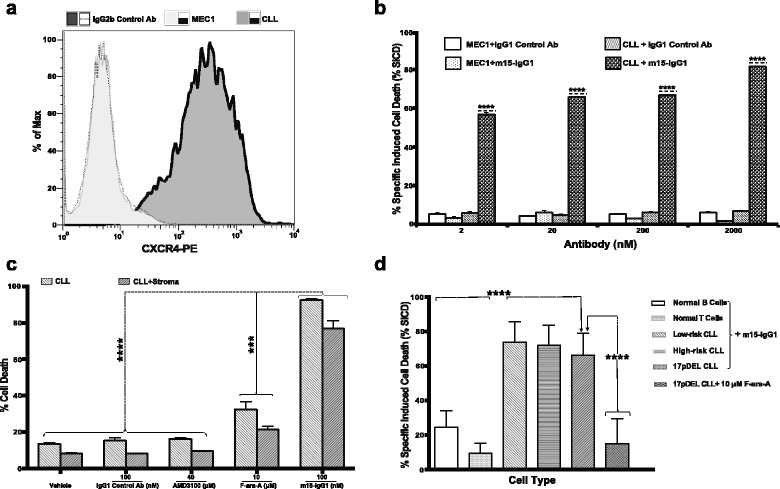
CXCR4 antibody-induced cell death is dependent on CXCR4-expression and independent of CLL disease risk factor or stromal presence. **a** CXCR4 expression profiling was done using an anti-CXCR4 antibody for staining in the MEC1 cell line and primary CLL-B cells from a representative patient, followed by analysis using flow cytometry. The CXCR4 expression is presented in ∆MFI. **b** MEC1 and CLL-B cells were treated with different concentrations of m15-IgG1 (2–2000 nM) or IgG1 control antibody, for 48 h followed by flow cytometry analysis to determine % SICD. Samples were tested in duplicates, with the mean and standard deviation shown for each group. **c** The CLL-B cells derived from a CLL patient were treated with either F-ara-A (3 and 10 μM), AMD3100 (4 and 40 μM), IgG1 control antibody, or m15-IgG1 antibody, in presence or absence of stroma NK-tert cells, for 48 h followed by analysis using flow cytometry. The results of samples analyzed in duplicates with the mean ± SD are shown for each group. **d** Primary leukemia CLL-B cells were obtained from CLL patients, with high-risk or low-risk phenotypes, or carrying *TP53* 17pDel mutation (*n* = 10 per group). Normal B and T lymphocytes were obtained from healthy donors (*n* = 10 per group). The % SICD was determined after treatment with 100 nM of m15-IgG1 for 48 h. The figure shows the mean ± SD for % SICD in different cell types. Statistical significance was determined using Bonferroni’s correction test for multiple comparison tests, where *, **, ***, and **** represent *p* < 0.05; *p* < 0.01, *p* < 0.001, and *p* < 0.0001, respectively

m15-IgG1 was evaluated for its ability to trigger cell death upon binding to primary CLL-B cells expressing CXCR4 or to the MEC1 (CLL) cell line, which has no detectable CXCR4 expression (ΔMFI = 0.01) (Fig. 3a). Cells were incubated with increasing concentrations of m15-IgG1 or control IgG1 antibody and analyzed for cell death using flow cytometry. CLL-B cells underwent cell death upon treatment with m15-IgG1 (2–2000 nM) in a dose-dependent manner, while MEC1 cells did not show evidence of cell death, even in presence of high concentrations of the antibody (Fig. 3b), indicating that the CXCR4 antibody cell death is CXCR4 expression dependent.

### The CXCR4 antibody induces cell death in spite of the presence of stromal cells

To determine whether m15-IgG1 could induce cell death in presence of stromal cells, CLL-B cells were cultured with stroma-NK-tert cells in presence of increasing concentrations of m15-IgG1 and cell death was evaluated after 48 h. F-ara-A, an agent that inhibits DNA synthesis and is a cornerstone for the treatment for CLL patients [36] as well as AMD3100, a CXCR4 small molecule inhibitor [37], were evaluated for comparison. m15-IgG1 induced cell death of leukemia cells cultured either alone or in presence of stromal cell support (stroma NK-tert cells), demonstrating the ability of the antibody to induce cell death in presence of stromal cells (Fig. 3c). Similar results were observed for various B-CLL patients (Additional file 3: Figure S3).

Moreover, m15-IgG1 was more potent at inducing cell death than F-ara-A (*p* < 0.0001) in CLL-B cells (Fig. 3c). AMD3100, which binds and inhibits signaling through CXCR4, did not induce cell death in CLL-B cells (Fig. 3c), indicating that binding and inhibition of the CXCR4 pathway is not sufficient to trigger cell death.

Next, we sought to determine whether the Fc constant region or backbone of the CXCR4 antibody played a role in the ability to induce B-CLL cell death. We compared m15-IgG1 to m15-IgG4, which is an antibody with the same antigen-binding regions from m15-IgG1, cloned in a human IgG4 constant region. Results from these studies (Additional file 4: Figure S4) demonstrate that both antibodies are capable to induce cell death in CLL-B cells to similar degrees, with no significant difference between m15-IgG1 and m15-IgG4, cultured alone or co-cultured with stroma cell support (*p* > 0.05). This indicates that the antibody constant region does not play a role in this cell death mechanism.

### The CXCR4 antibody induces B-CLL cell death independently of CLL risk factor and spares normal B and T lymphocytes

In addition to the high-risk and low-risk prognosis markers ZAP70 and IgVH, the 17p deletion in the *TP53* gene is also considered a strong independent adverse prognostic factor for survival and is associated with the short median treatment-free survival, in CLL patients with CLL [38].

To evaluate the ability of the CXCR4 antibody to induce cell death in leukemia cells from CLL patients with high risk (CLL-HR), low risk (CLL-LR), as well as with *TP53* 17pDel status, we treated samples from patients with these various genetic backgrounds with m15-IgG1 and evaluated cell death after 48 h of culture. Similar levels of cell death were induced by m15-IgG1 in CLL-LR, CLL-HR, *TP53wt*, or *TP53mut*/Del(17p) patient samples (Fig. 3d). This indicates that low-risk, high-risk, or *TP53* statuses are not factors for sensitivity to m15-IgG1. Moreover, m15-IgG1-induced cell death was significantly higher than that with F-ara-A (*p* < 0.0001), which is known to be a *TP*53-dependent chemotherapy agent [39, 40]. F-ara-A did not induce cell death in *TP53mut*/Del(17p) even at supra-physiological concentrations (>3 μM).

Importantly, m15-IgG1 induced significantly lower levels of cell death in normal B and T lymphocytes compared with CLL samples (*p* < 0.0001) (Fig. 3d). These data suggest that m15-IgG1-induced cell death is dependent on the level of CXCR4 expressed in the cells.

To characterize the kinetics of cell death induced by the CXCR4 antibody, a washout experiment was carried out, where CLL-B cells were incubated with m15-IgG1 (200 nM) for increasing lengths of time, after which the antibody was washed out. Readouts were performed 48 h post-treatment initiation. Cell death was observed as early as 3 h and continued to increase until 48 h after m15-IgG1 was removed (Additional file 5: Figure S5). This effect was also independent of presence of stromal cells.

### The CXCR4 antibody synergizes with CLL standard of care agents

**Fig. 4:**
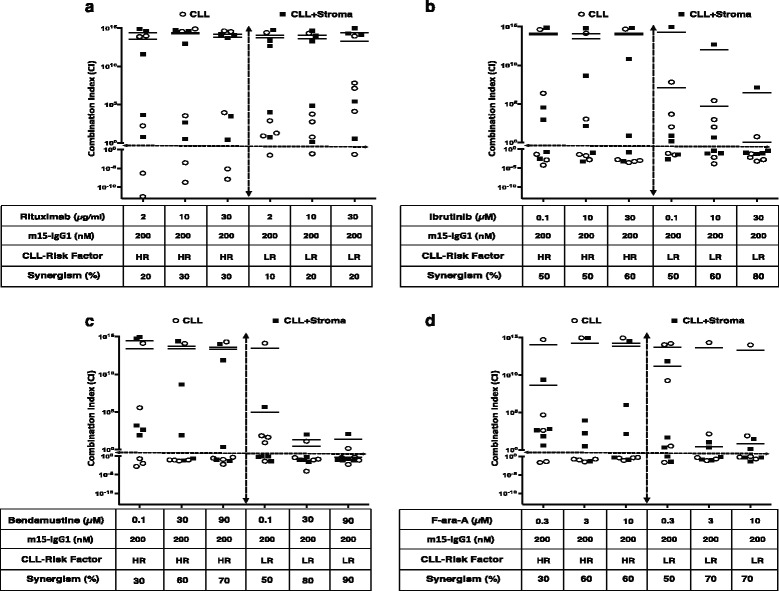
Cell death synergism of m15-IgG1 antibody with components of standard of care (SOC) in CLL. The primary leukemia CLL-B cells were incubated either alone or with stroma-NK-tert cells and treated with m15-IgG1 (200 nM) and three different concentrations of SOC agents **a** rituximab (1, 3, and 10 μg/mL), **b** ibrutinib (0.1, 10, and 30 μM), **c** bendamustine (0.1, 30, and 90 μM), and **d** fludarabine (F-ara-A, 1, 3, and 10 μM). Treatments were performed with each agent alone or in combination. The % cell death was used to calculate the median effect of the combinatorial effect of m15-IgG1 with each different SOC agent. The synergism between m15-IgG1 and SOCs was expressed as a combination index (CI), which uses the definitions: additive (CI = 1), synergistic (CI < 1), and antagonistic (CI > 1). The data were analyzed. The empty circle symbols (**O**) denote CLL cells alone, while solid black symbols (■) denote CLL cells co-cultured with stroma-NK-tert cells

To determine if the CXCR4 antibody could offer additional benefit to available therapies, the m15-IgG1 antibody was evaluated in combination with SOC agents currently used in the treatment of CLL patients in the clinic. The percent cell death combinatorial effect was evaluated in both high-risk (HR) and low-risk (LR) CLL patient cells, in presence or absence of stromal cell support (Fig. 4). Overall, the presence of stromal cells did not appear to be a significant factor in the ability of m15-IgG1 to synergize with CLL SOC agents. Rituximab, an anti-CD20 antibody [41, 42], was tested at 2, 10, and 30 μg/mL. Synergistic cell death responses, in the range of 10–30%, were observed for both low- and high-risk patients, for all rituximab doses tested (Fig. 4a). Ibrutinib (IBM) or imbruvica, a BTK inhibitor [43], was tested at 0.1, 10, and 30 μM, and synergism was observed in 50–60% of high-risk patients and 50-80% of low-risk patients samples (Fig. 4b). Bendamustine, a chemotherapy agent derived from nitrogen mustard, commonly used in the treatment of CLL and lymphomas [44], was tested at 0.1, 30, and 90 μM. Synergistic effects were noted in 30–70% of high-risk samples and in 50–90% of low-risk patients (Fig. 4c). F-ara-A was tested at 0.3, 3, and 10 μM doses. Synergistic effects were observed in 30–60% of high-risk and in 50–70% of low-risk patient samples (Fig. 4d).

### m15-IgG1 and PF-06747143 induce similar CLL cell death

**Fig. 5:**
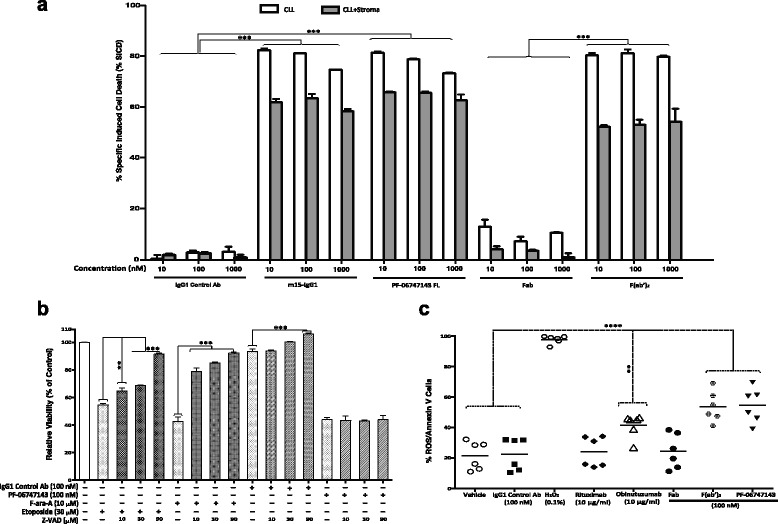
PF-06747143-induced cell death is bivalency-dependent, involves ROS, but not caspase activation. **a** Primary leukemia B cells obtained from a CLL patient were cultured alone or in presence of stroma-NK-tert cells after treatment with 10, 100, and 1000 nM of m15-IgG1, PF-06747143, or its Fab and F(ab’)_2_ forms, for 48 h. % SICD was measured by flow cytometry using CD19/CD5/Annexin V staining. The results of samples analyzed in duplicates with the mean ± SD are shown for each group. **b** CLL cells were incubated with PF-06747143 (100 nM), F-ara-A (10 μM), or etoposide (30 μM) for 48 h, either alone or in combination with different concentrations of a pan-caspase inhibitor, Z-VAD-FMK (Z-VAD) (10, 30, 90 μM). CLL cell death was analyzed by flow cytometry. **c** CLL-B cells (*n* = 6 per group) were incubated for 48 h with the intact CXCR4 antibody PF-06747143, the Fab and the F(ab’)_2_ forms of the antibody, the CD20 antibodies rituximab or obinutuzumab, or the IgG1 control antibody. H_2_O_2_ was used as a positive control. ROS production and cell dealth were analyzed by co-staining for ROS and CD19^+^/CD5^+^/Annexin V, respectively. The figure shows the individual data points for each group, and horizontal lines represent the mean of each group. Statistical significance was determined using Bonferroni’s correction test where *, **, ***, and **** represent *p* < 0.05; *p* < 0.01, *p* < 0.001, and *p* < 0.0001, respectively

To establish if the humanized IgG1 CXCR4 antibody PF-06747143 and its parent antibody m15-IgG1 had comparable cell death activity, we performed a cell death study in leukemic B cells derived from B-CLL patient, comparing both antibodies. As shown in Fig. 5a, activity of both antibodies was very similar, with cell death induced at doses as low as 10 nM by both antibodies. Both antibodies induced similar degree of cell death, regardless of the presence of stromal cells.

Intact antibodies possess two binding regions, which allow for binding to two epitopes at the same time. To determine if binding to two epitopes (bivalency) was required for PF-06747143 to induce cell death, the following forms of the PF-06747143 were compared: bivalent full-length (PF-06747143 FL), bivalent with no Fc region (F(ab’)_2_), and monovalent (Fab) (Fig. 5a). Comparable cell death (SICD) activity for the F(ab’)_2_ form of PF-06747143 antibody and the intact antibody (FL) was observed, while the Fab form of the antibody did not induce cell death. These results indicate that bivalency, or ability to bind to two epitopes at once, is required for PF-06747143 ability to induce cell death.

### PF-06747143 induction of cell death is caspase independent

To determine the mechanism through which PF-06747143 induced cell death, caspase activation was evaluated. PF-06747143 treatment of CLL-B cells did not induce significant activation of caspase 3, 8, and 9 in CLL-B cells (Additional file 6: Figure S6). In a follow-up experiment, Z-VAD-FMK (Z-VAD), an irreversible pan-caspase inhibitor [45, 46], rescued caspase-dependent apoptosis in CLL-B cells after treatment with etoposide or F-ara-A, which are both known to induce caspase-dependent cell death. However, Z-VAD failed to rescue the CLL-B cells treated with PF-06747143, indicating that the mechanism of cell death induced by PF-06747143 is caspase independent (Fig. 5b).

### Reactive oxygen species (ROS) production is associated with PF-06747143-induced cell death

Antibody-induced non-apoptotic cell death in human lymphoma and leukemia cells had been previously shown to be mediated through a ROS-dependent pathway, named programmed cell death (PCD) [32]. This novel cell death mechanism does not rely on caspase activation and is dependent on homotypic cell adhesion triggered upon antibody binding, followed by actin redistribution, lysosome membrane permeabilization, and ROS activation. Since we showed that PF-06747143 induction of cell death is caspase independent and bivalency dependent, we hypothesized that PF-06747143 cell death mechanism might involve ROS activation. To test this hypothesis, CLL-B cells were treated with PF-06747143 or controls and evaluated for ROS production after 4 h of treatment. CLL-B cells treated with H_2_O_2_, the positive control, showed high levels of ROS compared to untreated control (****, *p* < 0.0001), as expected. Similarly, CLL cells treated with PF-06747143 showed a rapid increase in cell death and ROS production, compared to the untreated or to the IgG1 control Ab groups (****, *p* < 0.0001). Of note, PF-06747143 treated cells had ROS levels similar to that of the positive antibody control obinutuzumab (*p* > 0.05), which has been reported to induce PCD associated with increased ROS levels [32]. Rituximab and F-ara-A, which are not known to induce PCD, did not show a significant increase in ROS (Fig. 5c).

To determine if binding to two epitopes (bivalency) was required for PF-06747143 ability to induce cell death via ROS production, the bivalent full-length (FL), the bivalent deprived of Fc region (F(ab’)_2_), and the monovalent (Fab) forms of the antibody were evaluated. Comparable ROS activity was observed for the F(ab’)_2_ form of PF-06747143 antibody and the intact antibody (FL), while the Fab form did not induce cell death or ROS production (Fig. 5c). These results indicate that bivalency, or ability to bind to two epitopes at once, is required for PF-06747143 to induce cell death via a mechanism that involves ROS induction.

### PF-06747143 inhibits F-actin polymerization in CLL cells

**Fig. 6:**
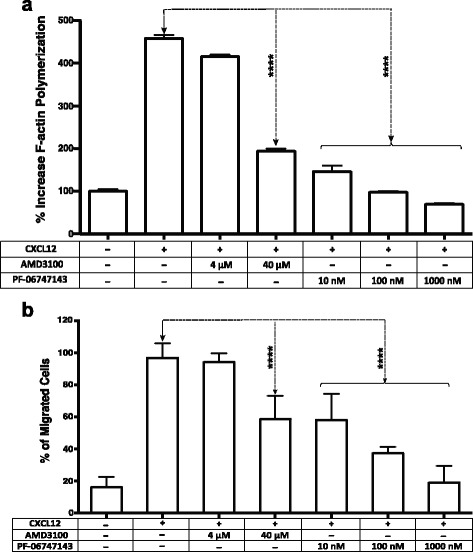
PF-06747143 inhibits CXCL12-induced tumor cell actin polymerization and migration. **a** B-CLL patient cells were treated with no compound (negative control), AMD3100 (4 and 40 μM), or PF-06747143 (10, 100, and 1000 nM) prior to stimulation with CXCL12 (90 nM) for 15 s. F-actin polymerization was measured using FITC-labeled phalloidin in CD19/CD5-pre-labeled CLL patient cells. All samples are plotted relative to the mean fluorescence intensity of the negative control group, without chemokine CXCL12, set to 100%. The results of samples analyzed in duplicates with the mean ± SD are shown for each group. **b** CLL patient primary cells were incubated with PF-06747143 (10, 100, 1000 nM) or AMD3100 (4 and 40 μM) for 1 h and loaded onto a transwell chamber and incubated for 2 h in the presence of CXCL12 (12 nM) or media control. Cells that migrated to the lower chamber were enumerated using flow cytometry. The results of samples analyzed in duplicates with the mean ± SD are shown for each group. Statistical significance was determined using Bonferroni’s correction test

Actin polymerization, or cytoskeletal reorganization, is a surrogate marker of cancer cell migration and metastatic potential, induced by the interaction of CXCR4 with CXCL12 [47]. Therefore, we sought to characterize PF-06747143 role in CXCL12-induced actin polymerization in CLL-B cells. Stimulation with CXCL12 induced an average increase in actin polymerization of 450%, relative to baseline (100%) (Fig. 6a). PF-06747143 significantly inhibited actin polymerization in a dose response-dependent manner. PF-06747143 at 100 and 1000 nM inhibited CXCL12-induced F-actin polymerization to levels below baseline at 85% and 75%, respectively (****, *p* < 0.0001). The small molecule inhibitor of CXCR4, AMD3100, was less potent than PF-06747143 in this assay, with very limited activity at 4 μM, which is its clinically relevant dose. At 40 μM, it showed moderate inhibition of 180%, which was similar to the inhibition observed at the lowest dose of PF-06747143 (10 nM) (Fig. 6a).

### PF-06747143 inhibits migration of CLL patient-derived cells

Since PF-06747143 was shown to be a potent inhibitor of cytoskeletal organization, we evaluated its ability to inhibit CLL-B cell migration driven by its ligand, CXCL12, using a transwell migration assay. CXCL12 induced chemotaxis of CLL-B cells, with an average increase >500% over baseline (Fig. 6b). PF-06747143 (10 nM–1 μM) significantly inhibited cell migration in a range from 40 to 80%, relative to CXCL12 induction only (****, *p* < 0.0001). The effect was dose dependent. CLL-B cells treated with AMD3100 (40 μM) had similar migration inhibition activity as PF-06747143 lowest dose (10 nM), but no significant effect was observed with AMD3100 at 4 μM (Fig. 6b).

### PF-06747143 induced CLL cell death via Fc region-mediated cytotoxicity (ADCC and CDC)

Therapeutic antibodies may rely on their constant region (Fc domain) ability to induce target cell killing via immune-mediated effector functions, such as ADCC and CDC to achieve efficacy. Human IgG1 and IgG3 antibodies have Fc domain sequences that can mediate potent effector functions, while human IgG4 and IgG2 antibodies display little or no effector function [48]. PF-06747143 is a humanized IgG1 antibody; therefore, ADCC and CDC studies were performed to characterize the antibody Fc-driven cytotoxic activity on CLL patient cells.

**Fig. 7:**
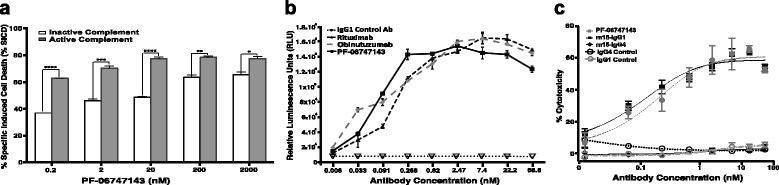
PF-06747143 induces CLL-B cell killing by ADCC and CDC. **a** CDC assay was performed by treating CLL-B cells (1 × 10^6^/mL) with PF-06747143, in presence of complete or heat-inactivated 5% human serum. The cells were incubated for 4 h and the cytotoxicity was determined using flow cytometry with CD19/CD5/Annexin V staining. **b** The ADCC assay in patient B-CLL cells was performed using 1:1 ratio of the target/effector cell (T/E) and incubated for 6 h at 37 °C. The IgG1 control antibody, PF-06747143, and rituximab were tested in a 1:3 titration curve, ADCC activity was determined using Bio-Glo™ luciferase assay, and the luminescence results are expressed in relative light units (RLU). The samples were analyzed in duplicates with the mean and SD shown for each group. The data was analyzed using Prism 4 GraphPad software. **c** ADCC activity was evaluated in JVM-13 tumor cells by incubating the cells with PF-06747143, m15-IgG1, m15-IgG4, or respective negative control antibodies for 4 h, in the presence of NK92 158V effector killer cells (effector/target cell ratio 10:1). Cell lysis was measured by ToxiLight bioluminescent cytotoxicity assay. Experiments were performed in quadruplicates with the mean ± SEM shown for each group

CDC cell death mechanism depends on the interaction between active serum complement proteins with the Fc region of the antibody, upon binding to the target cells. To evaluate if PF-06747143 could induce CDC in CLL-B cells, it was tested in the presence of active or inactive serum protein. As shown in Fig. 7a, PF-06747143 significantly (*p* < 0.0001) increased cytotoxicity in presence of active complement, relative to inactive complement, and this response was antibody dose dependent.

In the case of ADCC, effector cells bind to the Fc region of the antibody, when bound to target cells, and trigger cell lysis. To characterize the Fc-mediated ADCC activity of PF-06747143, CLL-B cells were incubated with the antibody in presence of effector cells. Two therapeutic antibodies, rituximab and obinutuzumab, that bind to CD20 and rely on ADCC as their main mechanism of action were used as positive controls. As expected, rituximab and obinutuzumab induced ADCC of CLL-B cells to high levels. PF-06747143 had comparable ADCC activity to that of the CD20 antibodies, inducing significant ADCC, when compared to the negative IgG1 control antibody (Fig. 7b). Taken together, these data demonstrate that PF-06747143, a humanized IgG1 antibody, has strong Fc-driven cytotoxic-dependent activity, leading to elimination of CLL-B cells.

To evaluate the role of the human IgG backbone in the ADCC activity of a CXCR4 antibody, we compared PF-06747143, m15-IgG1, and m15-IgG4, generated by cloning of the m15 variable domain in a human IgG4 backbone. The ADCC assay was performed using the CLL tumor cells (JVM-13), in presence of NK92158V effector cells. The m15-IgG1 antibody showed strong cytotoxicity, when compared to no activity with the m15-IgG4 antibody (Fig. 7c). This is in agreement with the expected diminished ADCC activity of an IgG4 antibody. In addition, PF-06747143 exerted similar cytotoxicity to that of m15-IgG1, confirming that the parent antibody (m15-IgG1) and its humanized antibody (PF-06747143) have comparable ADCC functional properties.

### PF-06747143 is efficacious as a monotherapy and in combination with bendamustine in a CLL xenograft established in vivo model

**Fig. 8:**
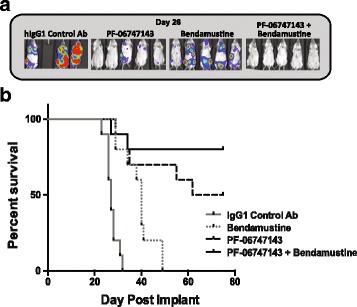
PF-06747143 inhibits tumor burden and increases survival as a monotherapy or in combination with bendamustine, in a disseminated CLL tumor model. JVM-13-Luc CLL cells were implanted intravenously (1 × 10^6^ cells) and allowed to spontaneously migrate and home in the bone marrow and lymph nodes for 19 days, when animals were randomized (*n* = 10/group). Animals were treated with IgG1 control or PF-06747143 antibodies at 10 mg/kg, subcutaneously, weekly, for six doses. Bendamustine was dosed at 30 mg/kg, intraperitoneally, on days 19 and 20, followed by another 2-day cycle 28 days later **a** Whole-body bioluminescence representative imaging showing bone marrow tumor burden on day 26. **b** Kaplan-Meier survival curve, using hind leg paralysis as endpoint

To determine if PF-06747143 is effective in eliminating CLL tumor cells in vivo, a disseminated model in which the tumor cells were implanted intravenously and migrate spontaneously to various sites in the body, including the BM and lymph nodes, was used (Fig. 8). Activity of PF-06747143 was evaluated as a monotherapy as well as in combination with bendamustine, a SOC agent approved for CLL treatment. Bioluminescence imaging performed on day 26 showed that PF-06747143 treatment as a monotherapy decreased tumor burden compared to hIgG1 control Ab and bendamustine groups. A combinatorial effect was observed when PF-06747143 was given with bendamustine (Fig. 8a). PF-06747143 strong tumor growth inhibition also resulted in significant survival benefit at the end of the study, day 75. The hIgG1 negative control Ab and bendamustine groups had median survival of 27 and 40 days, respectively, while PF-06747143 monotherapy group lived significantly longer, with median survival of 68.5 days (*p* < 0.0001 vs IgG1 control Ab and *p* < 0.0094 vs bendamustine) (Fig. 8b). The median survival for the PF-06747143 and bendamustine combination group was not reached by the last day of the study (day 75), demonstrating a combinatorial effect between these two agents. Taken together, these results show that PF-06747143 reduces BM and lymph node tumor burden and improves survival, as a monotherapy in a CLL disseminated tumor model. Moreover, its efficacy is improved in combination with bendamustine.

## Discussion

CXCR4 overexpression was shown to correlate with poor prognosis in CLL patients [23]. Activation of CXCR4 induces cell trafficking and homing of malignant cells to the BM and lymph nodes [49, 50], where CXCL12 is highly expressed, leading to retention of these cells in a microenvironment that provides growth signals, induces proliferation, and contributes to drug resistance, leading to poor prognosis and relapse [51]. In this study, we assessed the effect of inhibition of the CXCR4-CXCL12 pathway by a novel CXCR4 antagonist IgG1 antibody, PF-06747143, and its parental antibody m15-IgG1.

We showed that the surface expression of CXCR4 is at least tenfold higher in CLL patients than in normal T and B cells, in concordance with previous studies [52–54]. Similar CXCR4 expression levels were observed in high-risk or low-risk prognostic CLL patients. Importantly, both CLL risk groups had comparable sensitivity to CXCR4 antibody-induced cell death, while normal T and B cells, or the CXCR4-negative MEC-1 cell line, were largely spared. This indicates that the cell death mechanism is dependent on the level of CXCR4 surface expression. Notably, cells from CLL patients bearing the *TP53*mut/Del(17p) genotype, which is known to be resistant to F-ara-A therapy, were significantly sensitive to cell death induced by CXCR4 antibody treatment. This is of particular relevance in the treatment of refractory patients, who, in general, present with abnormal *TP53* status [55]. Furthermore, the similar levels of cell death observed in CLL-B cells cultured in presence or absence of stromal cell support suggest that the CXCR4 antibody has the potential to overcome the protection provided by the microenvironment. These findings may have significant clinical implications in the treatment of patients presenting with minimal residual disease in the BM and lymph nodes.

The CXCR4 antibody significantly synergized with CLL SOC agents (rituximab, ibrutinib, F-ara-A, and bendamustine), by increasing CLL cell death. Furthermore, we demonstrated that PF-06747143 improves survival as a monotherapy and its activity is increased when combined with bendamustine in the JVM-13 mouse xenograft disseminated staged model. These findings illustrate the potential of PF-06747143 to be used in combination with these agents in the clinic.

PF-06747143 blocked CXCL12-induced cytoskeletal changes and migration of primary CLL-B cells. Although to a lesser extent, these events were also inhibited by AMD3100, the CXCR4 small molecule inhibitor. However, the lack of cell death activity shown by ADM3100 in this study, and by CXCR4 peptide inhibitors described in the literature [4, 53, 56], suggests that just binding to CXCR4 and inhibiting CXCR4 signaling pathways is not sufficient to trigger this phenomenon. These differences may be explained by the observation that PF-06747143 induction of cell death required antibody bivalency. Bivalent binding is a property inherent to antibodies, due to their two binding regions, which are not a characteristic of small molecules or peptides. Other anti-CXCR4 antibodies, including ulocuplumab, LY2624587, and hz515H7, were also shown to induce tumor cell death upon binding to CXCR4 [23, 46, 57]; however, the role of antibody bivalency in this process was not described in these studies.

In further characterizing the mechanisms involved in the cell death triggered by PF-06747143, we demonstrated that this process did not involve caspase activation. The PF-06747143 caspase-independent cell death mechanism is similar to that described for ulocuplumab, an IgG4 CXCR4 antibody [46].

Recently, antibodies binding to CD20, CD74, CD47, or HLA-DR have been shown to directly induce programmed cell death (PCD), without involvement of caspases or the need for hyper-cross-linking of the antibody [32, 58]. This novel cell death mechanism is dependent on homotypic cell adhesion triggered upon antibody binding, followed by actin redistribution and ROS activation [59, 60]. Although it remains unclear which proximal events trigger PCD, the process results in loss of plasma membrane integrity and non-apoptotic cell death. A role for antibody bivalency in PCD has not been previously described; however, our results suggest that antibody bivalency might play a role in the initiating steps, by inducing homotypic cell-cell adhesion through binding to antigens expressed in adjacent cells simultaneously. We have also shown that PF-06747143 treatment generated ROS production in CLL-B cells, in association with cell death. The pattern of ROS production and cell death induced by PF-06747143 was similar to that observed with other antibodies shown to induce ROS-dependent cell death, such as CD20 (obinutuzumab), TAG-A1 [61], and 1D10 [62]. Taken together, our results suggest that upon binding to CXCR4 receptors in a bivalent manner, PF-06747143 triggers cell death through a caspase-independent and bivalency-dependent mechanism, similar to PCD.

In addition to signaling blockade and induction of bivalency-dependent cell death, PF-06747143 showed potent Fc effector-mediated ADCC and CDC activity in CLL-B cells. Of note, PF-06747143 and m15-IgG1 ADCC activity was significantly greater than that of the m15-IgG4 antibody in the ADCC assay. Similarly to m15-IgG4, ulocuplumab, which is a human IgG4 CXCR4 antibody, was recently reported to have no Fc-driven cytotoxic activity [46, 57]. The lack of Fc effector function-driven cytotoxicity observed for the IgG4 antibodies is expected, based on the human IgG4 inherently lower affinity for the proteins involved in the process [63]. We also showed that PF-06747143 cytotoxic activity was comparable to that of obinutuzumab and rituximab [64]. The importance of ADCC or CDC activity has been clinically demonstrated for obinutuzumab and rituximab, as well as other antibodies successful therapeutic IgG1 antibodies approved for the treatment of cancers, including alemtuzumab (CD52), trastuzumab (HER2), cetuximab (EGFR), and daratumumab (CD38) [48, 65].

Therapeutic CXCR4 antagonists currently available lack desirable potency, cytotoxicity, safety, or adequate exposure for prolonged treatment. The small molecule AMD3100 approved for stem cell mobilization in autologous transplantation has significant safety issues that limit its chronic use [16]. In addition, we showed that AMD3100 does not induce significant cell death in B-CLL cells. Peptide antagonists of CXCR4 have been recently evaluated in clinical trials as mobilizing agents. LY2510924 was evaluated as a single agent in a phase 1 dose escalation trial in advanced metastatic cancers [66], and BKT140 (BL8040/TN14003) was evaluated in a phase 1 clinical trial in multiple myeloma (MM) patients [19]. Treatment with both peptides induced rapid mobilization of stem cells but failed to reduce tumor burden. In addition, as for peptide therapeutics in general, they had short half-lives and required frequent administration, which makes peptides a challenging modality for sustained treatment [19, 66, 67]. The CXCR4 antagonist humanized IgG4 antibody, ulocuplumab, was recently evaluated in phase 1 clinical trials [20–22] and, as expected for an antibody, it exhibited a longer half-life than that of small molecules and peptides. In contrast to small molecule and peptide inhibitors of CXCR4, ulocuplumab, as well as another IgG4 CXCR4 antibody, LY26245587, and an IgG1 CXCR4 antibody hz515H7 [68], can induce tumor cell death via a mechanism reminiscent of PCD, similarly to PF-06747143.

However, the IgG4 antibodies do not induce tumor cell death via ADCC or CDC [23, 46], as expected for human IgG4 antibodies. PF-06747143 and hz515H7 are the first IgG1 CXCR4 antibodies to be described. A key role for Fc effector functions ADCC and CDC was demonstrated when a mutation in the Fc region of hz515H7, abrogating the Fc effector function, resulted in significantly decreased efficacy in a mouse tumor model [68]. Taken together, these data suggest that the CXCR4 antibody Fc effector cytotoxic functions, ADCC and CDC, play a key role in vivo and they may contribute to efficacy enhancement in the clinic.

## Conclusions

The novel CXCR4 antagonist IgG1 antibody PF-06747143 binds to CXCR4 with high affinity and blocks CXCL12-induced mechanisms including calcium flux and cell migration. The CXCR4 antibody mediates CLL-B cell death via a bivalency-dependent mechanism, involving generation of reactive oxygen species (ROS), with no caspase activation requirement, reminiscent of PCD. Moreover, PF-06747143 induces B-CLL cell death regardless of patient prognostic risk factor or the presence of stromal cells, indicating that it may be valuable in the treatment of resistant disease. PF-06747143 synergizes with CLL SOC agents such as bendamustine, rituximab, fludarabine, and ibrutinib. Moreover, in a CLL xenograft tumor model, PF-06747143 causes tumor growth inhibition, with increased survival, both as a monotherapy and in combination with bendamustine. Differently from other CXCR4 antagonists in the clinic, PF-06747143 induces potent cell death via Fc-driven cytotoxicity, through ADCC and CDC. Taken together, our data support the development of PF-06747143 for the treatment of CLL patients.

## Additional files

Additional file 1: Figure S1. CXCR4 expression profiling in different cell lines. CXCR4 expression was performed using the 2B11 CXCR4 antibody clone for surface staining of MEC1, K562, Raji, Ramos, Jurkat, Namalwa, and JeKo-1 cell lines followed by analysis of samples using flow cytometry. The CXCR4 expression is presented as ∆MFI. (PDF 528 kb)

Additional file 2: Figure S2. PF-06747143 and its Fab and F(ab’)2 forms block CXCL12-induced calcium flux. The calcium flux assay was performed in human T cell leukemia Jurkat cells incubated with PF-06747143 full-length (FL), PF-06747143-Fab, PF-06747143 F(ab’)2, or isotype control IgG1 antibody in presence of CXCL12 at 8 nM. For adequate comparison between the different forms of the antibody, their concentrations were adjusted relative to their antigen-binding site numbers. Experiment was performed in quadruplicates. The mean intracellular calcium concentration is shown in relative fluorescence units (RFU). Bars denote standard error of the mean (SEM). (PDF 389 kb)

Additional file 3: Figure S3. The PF-06747143 parent IgG1 antibody (m15 IgG1) induces cell death and this activity is similar in HR and LR CLL patients. Primary CLL-B cells derived from CLL patients were incubated either alone (*n* = 10) or co-cultured with stroma-NK-tert cells (*n* = 10) and treated with vehicle, IgG1 control Ab, or m15-IgG1 antibody for 48 h. Cell death was measured using CD19/CD5/Annexin V staining followed by flow cytometry analysis. The data is derived from five high-risk (HR) and five low-risk (LR) CLL patients. The HR patients are presented with solid symbols (•) and LR patients denoted with hollow symbols (○). The individual data points for each group are shown. The horizontal lines represent the mean for each group Statistical comparisons were performed using Bonferroni’s correction test. (PDF 744 kb)

Additional file 4: Figure S4. m15-IgG1 and m15-IgG4 have similar cell death activity in HR and LR CLL patients, in presence or absence of stromal cells. The primary CLL-B cells derived from CLL patients were incubated either alone (*n* = 4) or co-cultured with stroma-NK-tert cells (*n* = 4) and treated with vehicle, m15-IgG1, m15-IgG4, IgG1 control antibody, or IgG4 control antibody for 48 h. Cell death was measured using CD19/CD5/Annexin V staining followed by flow cytometry analysis. The data is presented as % specific induced cell death (% SICD). The data shown is derived from two high-risk (HR) and two low-risk (LR) CLL patients. The HR patients are presented with solid symbols (•) and LR patients denoted with hollow symbols (○). The individual data points for each group are shown. The horizontal lines represent the mean for each group. Statistical comparisons were performed using Bonferroni’s correction test. (PDF 1032 kb)

Additional file 5: Figure S5. The CXCR4 antibody-induced CLL cell death increases over time, in presence or absence of stromal cells. In this washout experiment, patient CLL cells cultured alone or in presence of stroma-NK-tert cells and were treated with vehicle or m15-IgG1 antibody (200 nM) for 0.5, 2, 6, 12, 24, 36, and 48 h. Cell death was measured using CD19/CD5/Annexin V staining followed by flow cytometry analysis. The samples were tested in duplicates. Statistical comparisons were performed using Bonferroni’s correction test. (PDF 513 kb)

Additional file 6: Figure S6. m15-IgG1-induced LR or HR CLL-B cell death is independent of caspase activation. CLL-B cells were treated for 6 h with m15-IgG1 (1, 10, or 100 nM) or IgG1 control antibody. Caspases 3, 8, and 9 were measured using a colometric detection method. The data shown is derived from four high-risk (HR) and four low-risk (LR) CLL patients. The HR patients are denoted by triangles and LR patients denoted by circles. The individual data points for each group are shown. The horizontal lines represent the mean for each group. Statistical comparisons were performed using Bonferroni’s correction test. (PDF 915 kb)

## Abbreviations

ADCC: Antibody-dependent cell-mediated cytotoxicity
AML: Acute myeloid leukemia
CDC: Complement-dependent cytotoxicity
CLL: Chronic lymphocytic leukemia
CXCL12: C-X-C motif chemokine ligand 12
CXCR4: C-X-C chemokine receptor type 4
F-ara-A: Fludarabine
PCD: Programmed cell death
ROS: Reactive oxygen species
SICD: Specific induced cell death
SOC: Standard of care
