# Supplementary figures and images for: Targeting the CXCR4 pathway using a novel anti-CXCR4 IgG1 antibody (PF-06747143) in chronic lymphocytic leukemia
Source: J Hematol Oncol. 2017 May 19;10:112. doi: 10.1186/s13045-017-0435-x (PMC5438492; doi:10.1186/s13045-017-0435-x)

# Supplementary Figure 1.

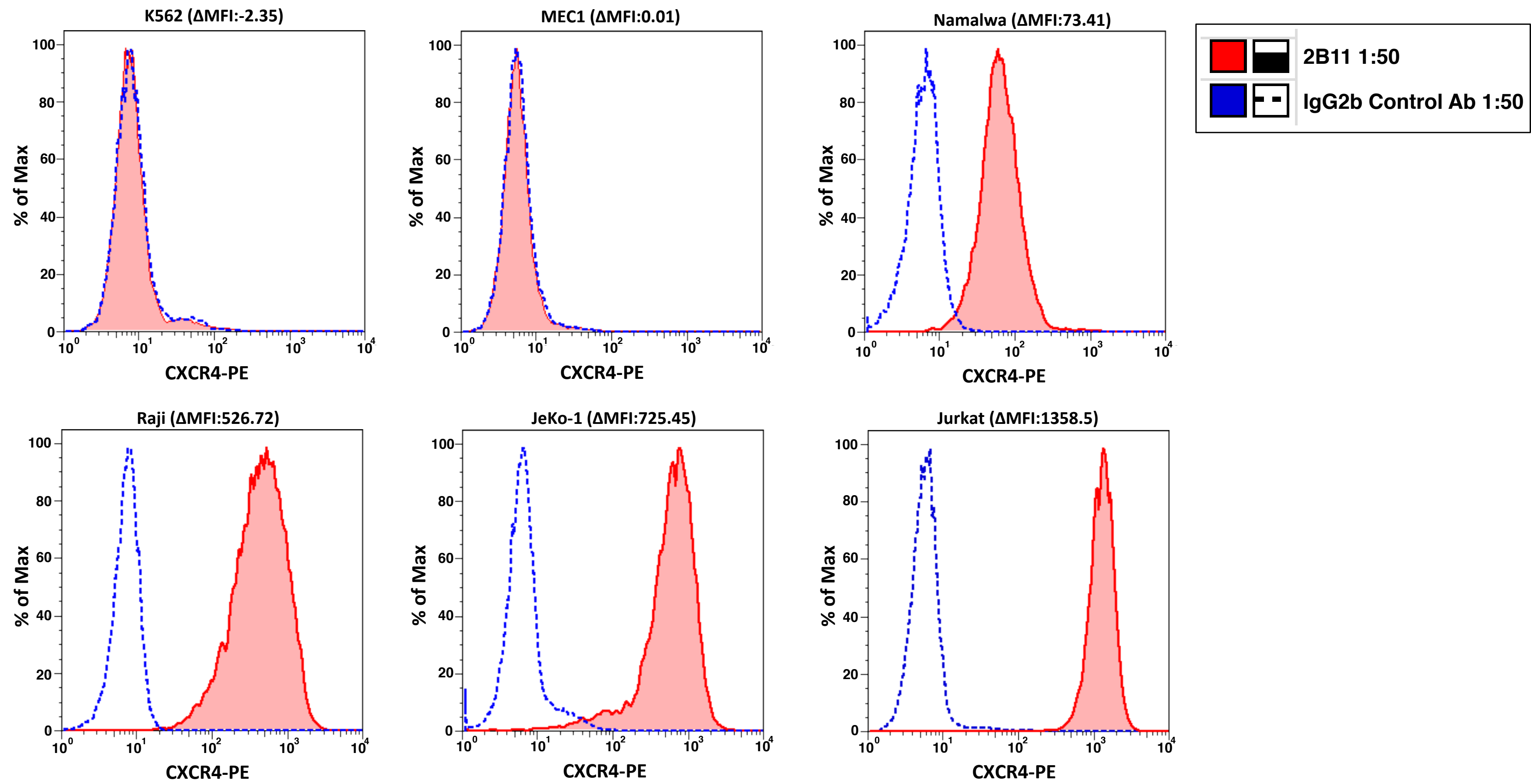

Supplement: Supplementary file 1 — CXCR4 expression profiling in different cell lines. CXCR4 expression was performed using the 2B11 CXCR4 antibody clone for surface staining of MEC1, K562, Raji, Ramos, Jurkat, Namalwa, and JeKo-1 cell lines followed by analysis of samples using flow cytometry. The CXCR4 expression is presented as ∆MFI. (PDF 528 kb) [file 13045_2017_435_MOESM1_ESM.pdf]

Supplementary Figure 2.

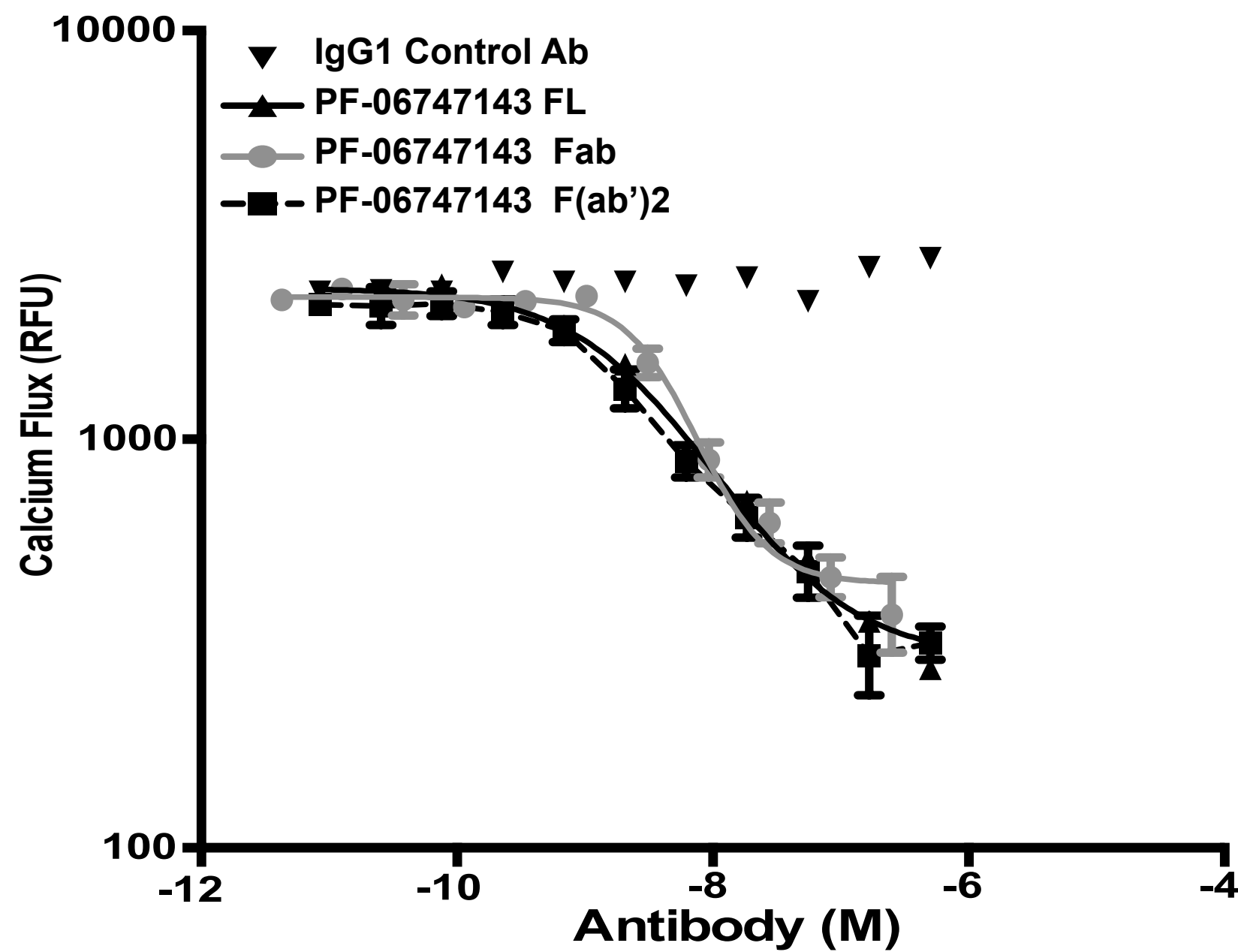

Supplement: Supplementary file 2 — PF-06747143 and its Fab and F(ab’)2 forms block CXCL12-induced calcium flux. The calcium flux assay was performed in human T cell leukemia Jurkat cells incubated with PF-06747143 full-length (FL), PF-06747143-Fab, PF-06747143 F(ab’)2, or isotype control IgG1 antibody in presence of CXCL12 at 8 nM. For adequate comparison between the different forms of the antibody, their concentrations were adjusted relative to their antigen-binding site numbers. Experiment was performed in quadruplicates. The mean intracellular calcium concentration is shown in relative fluorescence units (RFU). Bars denote standard error of the mean (SEM). (PDF 389 kb) [file 13045_2017_435_MOESM2_ESM.pdf]

Supplementary Figure 4

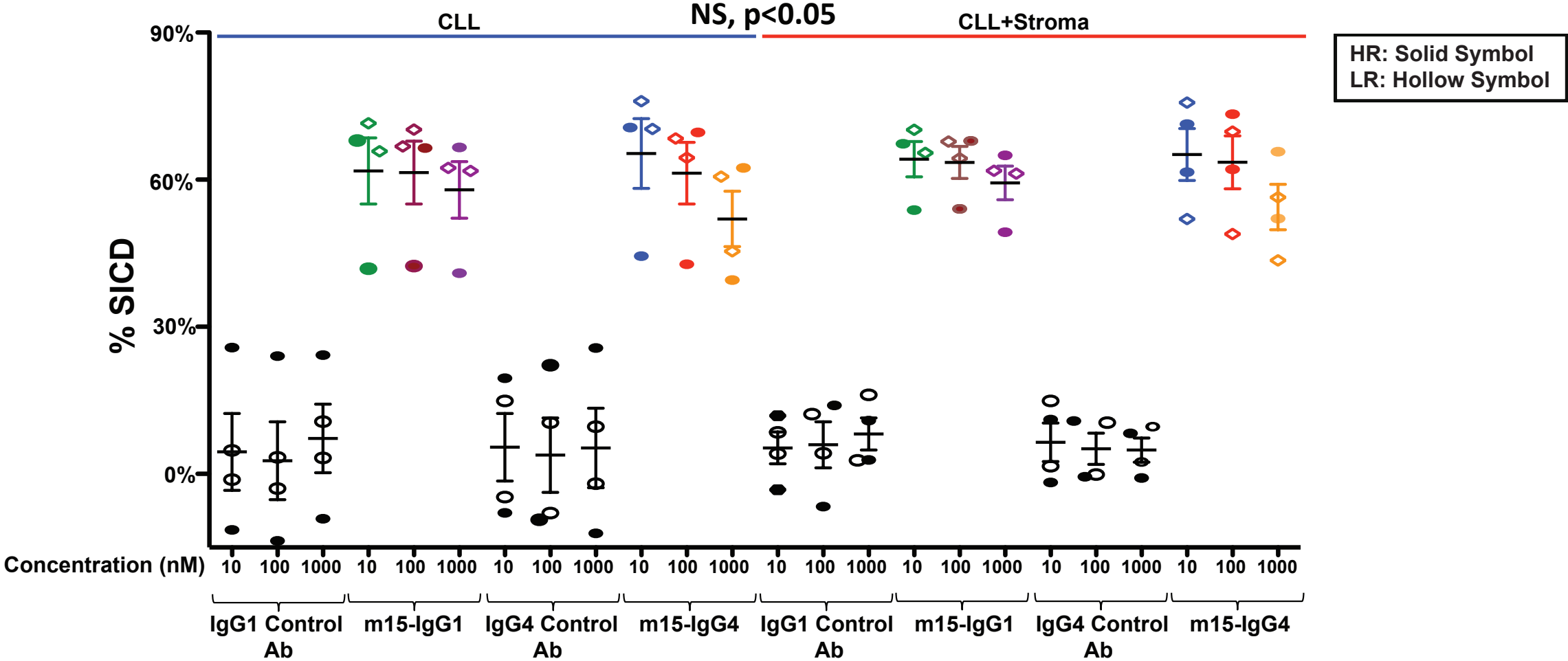

Supplement: Supplementary file 4 — m15-IgG1 and m15-IgG4 have similar cell death activity in HR and LR CLL patients, in presence or absence of stromal cells. The primary CLL-B cells derived from CLL patients were incubated either alone (n = 4) or co-cultured with stroma-NK-tert cells (n = 4) and treated with vehicle, m15-IgG1, m15-IgG4, IgG1 control antibody, or IgG4 control antibody for 48 h. Cell death was measured using CD19/CD5/Annexin V staining followed by flow cytometry analysis. The data is presented as % specific induced cell death (% SICD). The data shown is derived from two high-risk (HR) and two low-risk (LR) CLL patients. The HR patients are presented with solid symbols (•) and LR patients denoted with hollow symbols (○). The individual data points for each group are shown. The horizontal lines represent the mean for each group. Statistical comparisons were performed using Bonferroni’s correction test. (PDF 1032 kb) [file 13045_2017_435_MOESM4_ESM.pdf]

Supplementary Figure 5.

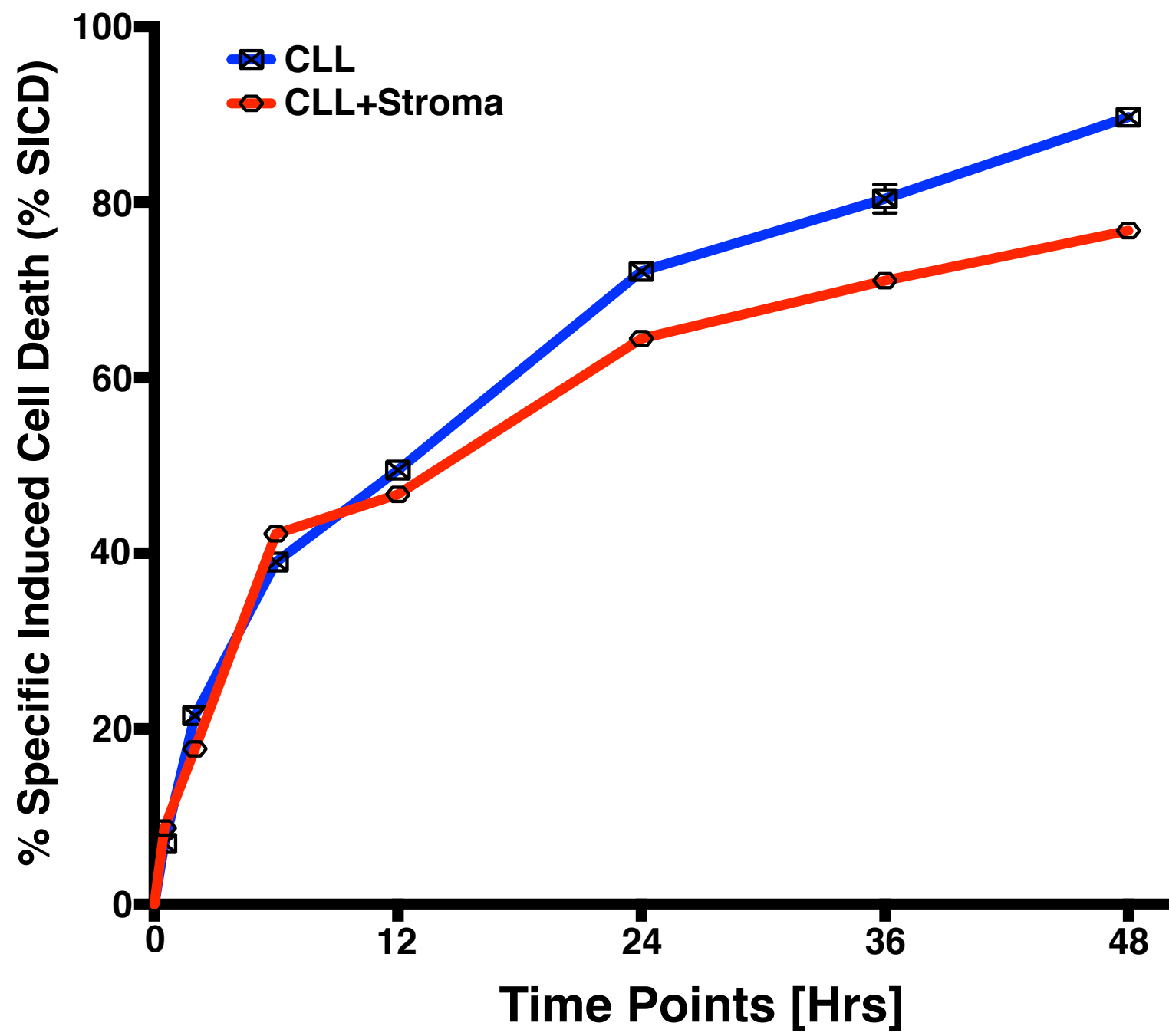

Supplement: Supplementary file 5 — The CXCR4 antibody-induced CLL cell death increases over time, in presence or absence of stromal cells. In this washout experiment, patient CLL cells cultured alone or in presence of stroma-NK-tert cells and were treated with vehicle or m15-IgG1 antibody (200 nM) for 0.5, 2, 6, 12, 24, 36, and 48 h. Cell death was measured using CD19/CD5/Annexin V staining followed by flow cytometry analysis. The samples were tested in duplicates. Statistical comparisons were performed using Bonferroni’s correction test. (PDF 513 kb) [file 13045_2017_435_MOESM5_ESM.pdf]
