# Supplementary material for: Targeting the CXCR4 pathway using a novel anti-CXCR4 IgG1 antibody (PF-06747143) in chronic lymphocytic leukemia
Source: J Hematol Oncol. 2017 May 19;10:112. doi: 10.1186/s13045-017-0435-x (PMC5438492; doi:10.1186/s13045-017-0435-x)
Supplement: Supplementary file 6 — m15-IgG1-induced LR or HR CLL-B cell death is independent of caspase activation. CLL-B cells were treated for 6 h with m15-IgG1 (1, 10, or 100 nM) or IgG1 control antibody. Caspases 3, 8, and 9 were measured using a colometric detection method. The data shown is derived from four high-risk (HR) and four low-risk (LR) CLL patients. The HR patients are denoted by triangles and LR patients denoted by circles. The individual data points for each group are shown. The horizontal lines represent the mean for each group. Statistical comparisons were performed using Bonferroni’s correction test. (PDF 915 kb) [file 13045_2017_435_MOESM6_ESM.pdf]

# Supplementary Figure 6.

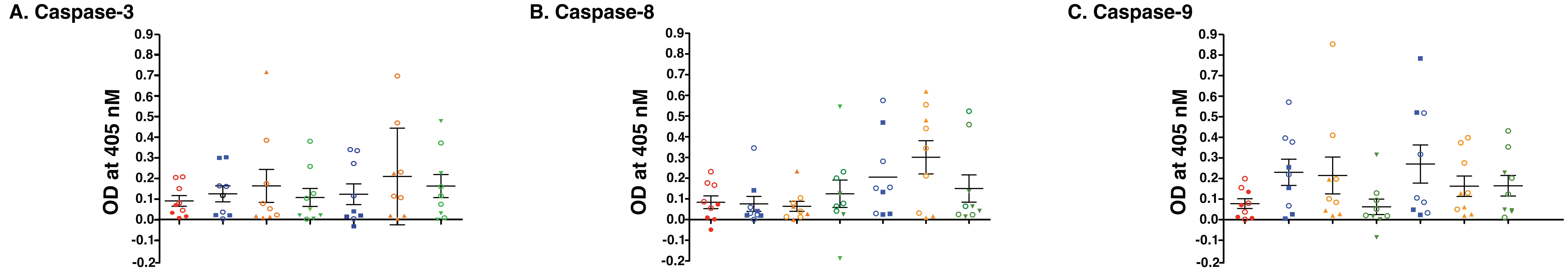


| IgG1 Control Ab (nM) | – | 1 | 10 | 100 | + | + | + |
|---|---|---|---|---|---|---|---|
| PF-06747143 | – | + | + | + | 1 | 10 | 100 |

| IgG1 Control Ab (nM) | – | 1 | 10 | 100 | + | + | + |
|---|---|---|---|---|---|---|---|
| PF-06747143 | – | + | + | + | 1 | 10 | 100 |

| IgG1 Control Ab (nM) | – | 1 | 10 | 100 | + | + | + |
|---|---|---|---|---|---|---|---|
| PF-06747143 | – | + | + | + | 1 | 10 | 100 |
