# Supplementary material for: Targeting the CXCR4 pathway using a novel anti-CXCR4 IgG1 antibody (PF-06747143) in chronic lymphocytic leukemia
Source: J Hematol Oncol. 2017 May 19;10:112. doi: 10.1186/s13045-017-0435-x (PMC5438492; doi:10.1186/s13045-017-0435-x)
Supplement: Supplementary file 3 — The PF-06747143 parent IgG1 antibody (m15 IgG1) induces cell death and this activity is similar in HR and LR CLL patients. Primary CLL-B cells derived from CLL patients were incubated either alone (n = 10) or co-cultured with stroma-NK-tert cells (n = 10) and treated with vehicle, IgG1 control Ab, or m15-IgG1 antibody for 48 h. Cell death was measured using CD19/CD5/Annexin V staining followed by flow cytometry analysis. The data is derived from five high-risk (HR) and five low-risk (LR) CLL patients. The HR patients are presented with solid symbols (•) and LR patients denoted with hollow symbols (○). The individual data points for each group are shown. The horizontal lines represent the mean for each group Statistical comparisons were performed using Bonferroni’s correction test. (PDF 744 kb) [file 13045_2017_435_MOESM3_ESM.pdf]

Supplementary Figure 3.

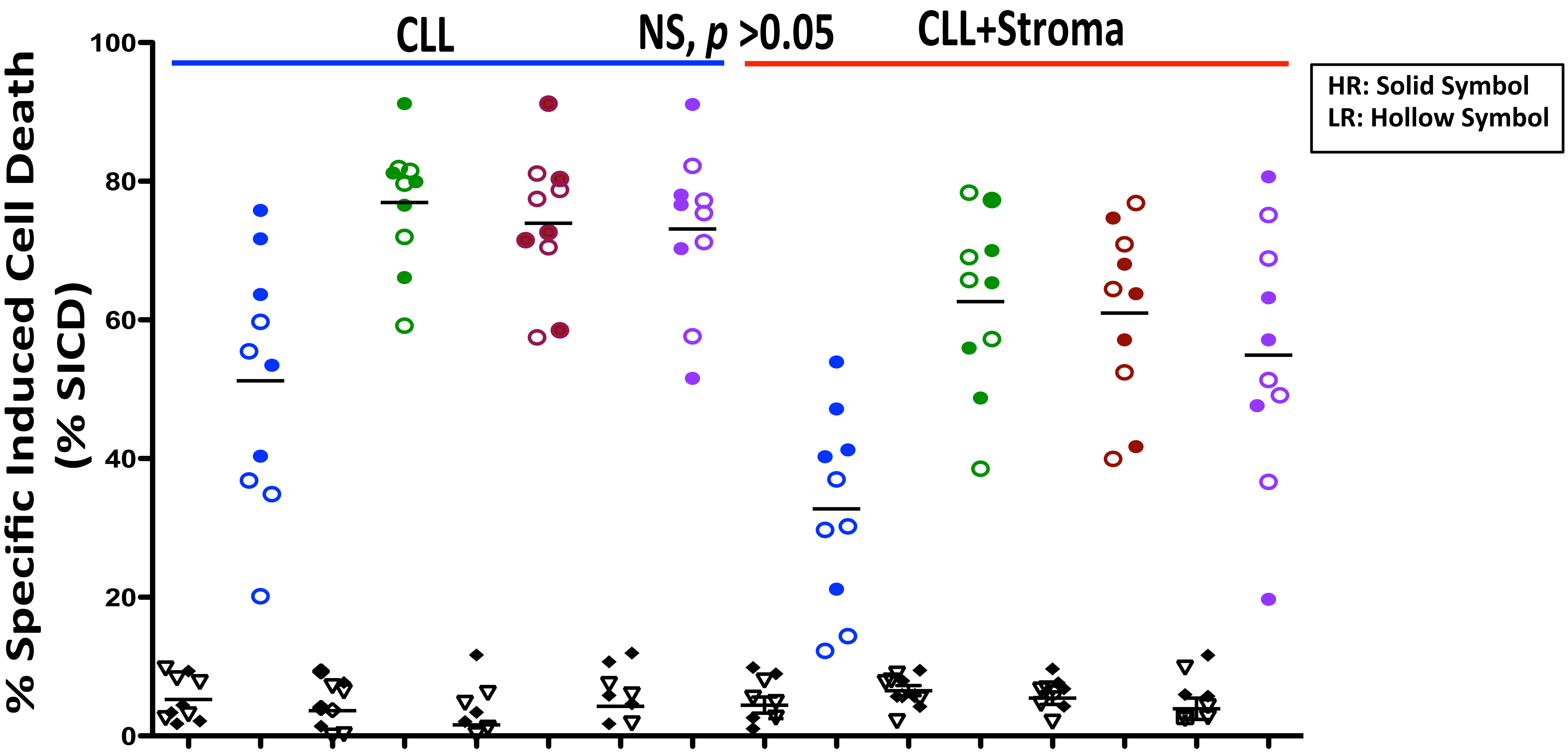

Antibody Concentration (nM)	1		10		100		1000		1		10		100		1000	
CLL B Cells	+	+	+	+	+	+	+	+	+	+	+	+	+	+	+	+
IgG1 Control Ab	+	-	+	-	+	-	+	-	+	-	+	-	+	-	+	-
m15-IgG1 Ab	-	+	-	+	-	+	-	+	-	+	-	+	-	+	-	+
Stromal Cells	-	-	-	-	-	-	-	-	+	+	+	+	+	+	+	+
